# Identifying and addressing social determinants of health to improve patient-centered care

**DOI:** 10.1017/cts.2024.511

**Published:** 2024-04-05

**Authors:** Neil Kalsi, Denai Gordon, Jenenne Geske

**Affiliations:** University of Nebraska Medical Center, Omaha, NE, USA

**Keywords:** Primary care, patient-centered, social determinants of health, self-reported, barriers to care

## Abstract

The purpose of this clinical improvement project was to instill a streamlined process of identifying social determinants of health (SDOH) in our clinic’s diverse patient population and provide resources that address these barriers to health and well-being. At each clinic visit, patients self-identified SDOH through an easy-to-use Social Assessment Form. Using an online database, Community Relay (CR), providers had access to location-based community resources. In addition to accomplishing the above-mentioned goals, we were left with a more well-rounded understanding of our patients. Unique struggles were identified and barriers to care were revealed, allowing for more patient-centered medical care.

## Introduction

Medical care plays a seemingly minuscule role in health outcomes when considering that the socioeconomic influences that patients experience daily affect as much as 80% of health outcomes [1]. The impact of social determinants of health (SDOH) on health outcomes is so great and well established that the Centers for Medicare and Medicaid Services (CMS) will require certain SDOH measures for inpatient reporting in 2024 [2] and reimburse for SDOH screening at Medicare wellness visits. Several other US government health agencies under the Department of Health and Human Services (DHHS) are increasing their focus to address SDOH due to the clear link of SDOH to health outcomes [2].

The concept of identifying these potential barriers to care is not novel. Screening for SDOH has been a common theme among groups looking to implement a more well-rounded approach to patient care. Numerous tools have been developed for the screening of SDOH in primary care [1]. Even our own organization’s electronic health record has an integrated screening tool. The issue is time. In a system where time is money and staffing is often short, providers are hesitant to add additional steps into their workflow. Unfortunately, many of the well-established screening tools are lengthy and time-consuming. A group of pediatricians developed a framework to address SDOH in the clinical setting which focuses on first assessing the needs of the community you serve and then developing a screening tool and identifying local resources. Next steps include a feedback loop of developing a workflow and eliciting feedback from clinic staff and patients [3]. A pilot looking into the feasibility of screening for SDOH found that initially 58% of clinicians thought they were too busy to add screening into their practice. However, by the end of the pilot that number had decreased to 21% [4]. Achieving buy-in from stakeholders is a potential barrier to implementing a process to screen and address SDOH. Furthermore, aligning with the goals of the organization at large can help with long-term success [3].

Moving from screening to addressing SDOH creates an additional time challenge, leading to more referrals to social work. Without an efficient implementation of a screening process, this key piece of medical history might be ignored.

We serve a diverse patient panel with patients of varying socioeconomic status, races/ethnicities, education level, and medical complexity. There was no standardized process to identify or address the SDOH that negatively affected the patients we serve. Identifying these often-overlooked variables is important; addressing them is arguably more important.

The goal of our study was to test a standardized SDOH screening tool that is easy to use and helps physicians identify the SDOH that are most prevalent in our patient population and to determine the feasibility of using a tool to address SDOH that arose from the screener.

Our research questions were as follows:

- Is it feasible to incorporate a SDOH screening questionnaire into our clinic workflow?
- Which SDOH most affect our clinic patients?

## Materials and methods

Patients of the two physician investigators at a Family Medicine clinic at a large, urban teaching hospital served as the initial pilot participants. The cross-sectional study pilot-tested a Social Assessment Form (SAF) designed for patients to identify SDOH that may have negative impacts on their health and to help connect patients with resources to address the issues identified by patients. A completed SAF was considered any SAF that the patient marked a SDOH with desire to discuss with provider, SDOH without desire to discuss with provider, or “I prefer not to complete this form.”

The SAF was developed as a companion to an online tool, Community Relay (CR) [5], by the Nebraska Health Network, a regional accountable care organization. CR is a database that allows users to find community resources for numerous goods and services, including transportation, housing, food, household goods, legal aid, employment, etc. Resources are filtered by zip code and further refined by specific patient demographics. Referrals are sent to selected programs who then contact the patient, or the resource information is printed and provided to the patient to contact on their own. Patients are also able to use this user-friendly tool to locate these community resources on their own phone or computer.

The SAF (supplementary material) was developed with categories and icons corresponding to those listed on the CR site. It was important for our screening form to be both easy to complete and interpret, with the goal of limiting disruptions to clinic workflow. Patients were asked to complete the voluntary SAF at each visit at the time of check-in and could indicate if they wanted to discuss their responses with the healthcare team. Based on the self-assessment, providers or rooming staff offered further discussion including the use of CR to provide information on community resources specific to patients’ needs.

Knowing the importance that clinic support staff would play in the success of the pilot, prior to implementation, staff were surveyed to assess current knowledge about SDOH, which SDOH affect our patients, and if they had feedback on how implementing a process to identify and address SDOH would affect the current clinic workflow. Training was then provided for use of the SAF and the CR website.

Descriptive statistics are reported addressing the number of patients who completed the SAF and summarizing their responses to SDOH that may impact their health.

## Results

Results of our pilot study verified that the SAF could be incorporated into the clinic workflow. We collected 598 SAFs, representing 598 encounters over a 9-month pilot period. We did not collect demographic information from survey respondents. However, the patient population of the clinic is 61% female. Approximately 22% of the clinic population is between the ages of 26 and 34 years, 31% between the ages of 35 and 54 years, and 16% between the ages of 55 and 65 years. Those over the age of 65 years account for 18% of the clinic population and those under the age of 26 years represent 13%. About 66% of the clinic population is White and 20% is Black or African American. Medicare and Medicaid make up 60% of the payor mix. The vast majority speak English, about 95%. The patient population of the study providers is similar to the clinic population.

**Figure 1.**
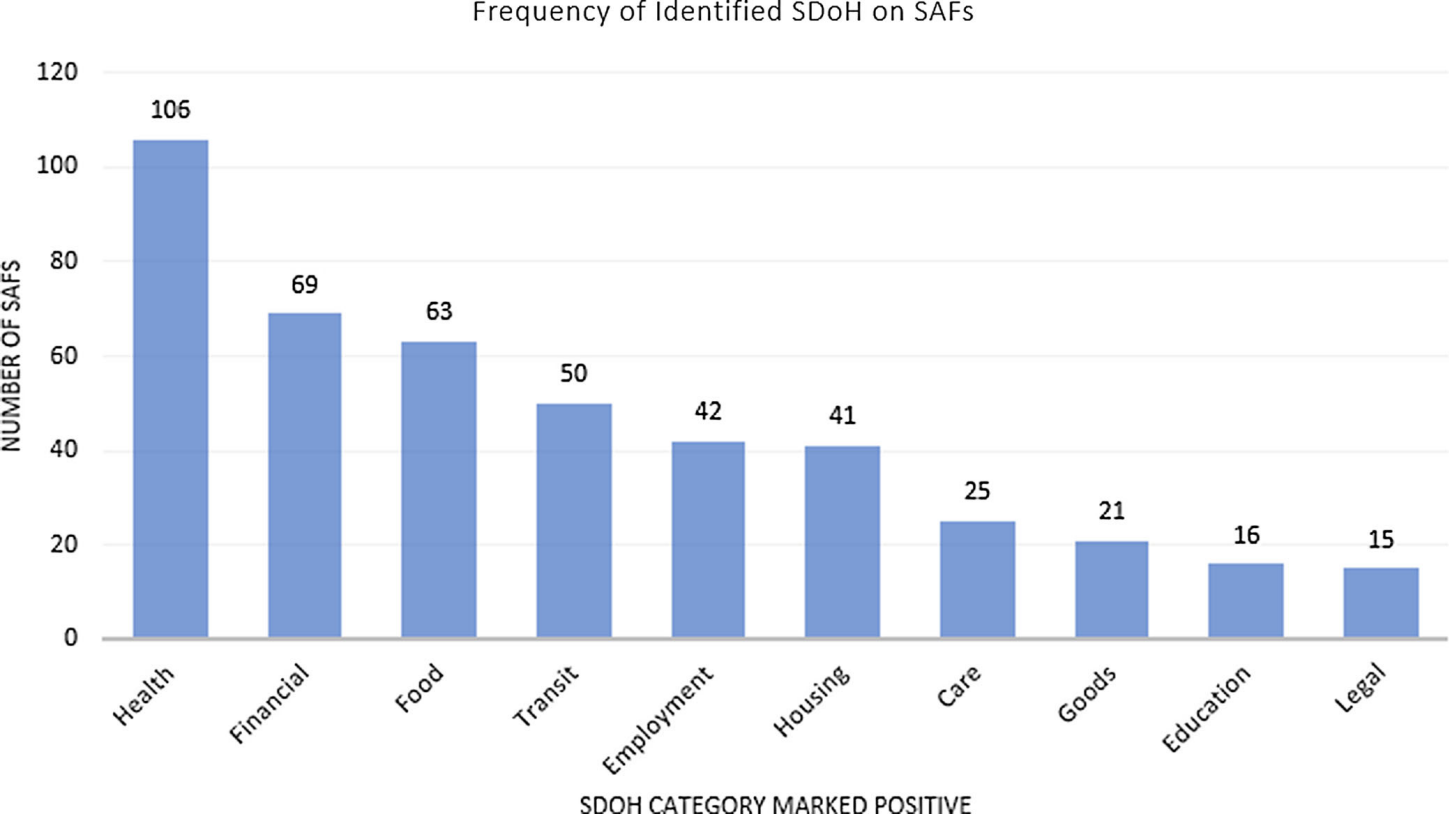
Frequency of social determinants of health (SDOH) categories indicated by patients. SAF = Social Assessment Form.

Of the 598 completed SAFs, 198 (33%) of them had at least one SDOH category marked. Of these 198 positive surveys, 38% indicated the patient did want to discuss their responses with their provider. The top five most frequently marked categories were health, financial, food, transit, and employment (Fig. 1).

During the pilot period, some patients found the “health” category on the SAF confusing. Persons completing the form were sometimes marking “health” simply to state their reason for visit. The health category was intended to capture issues regarding access to healthcare. We removed the “health” category from the data and found that 27% of surveys then had an SDOH marked. In 36% of these positive surveys, the patient marked they did want to discuss the SDOH with their provider [6,7].

**Figure 2.**
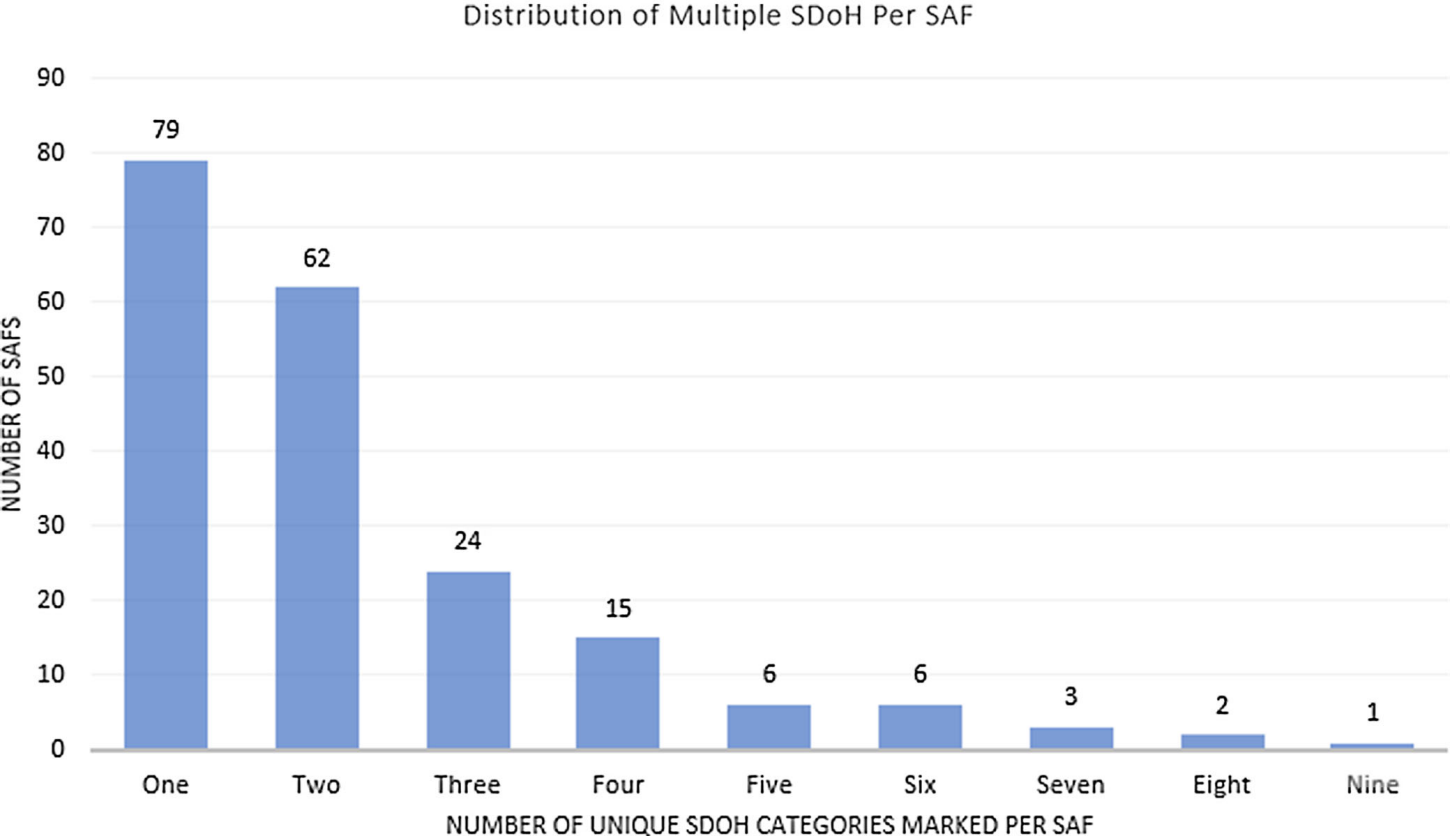
Distribution of multiple social determinants of health (SDOH) per Social Assessment Form (SAF).

A majority of 198 positive SAFs (63%) had more than one SDOH marked (Fig. 2). Fifty-six of the positive SAFs had three or more SDOH categories marked (28%).

In regard to the ease of the workflow of the SAF administration and review of CR, the study providers did not find the process cumbersome or overly time-consuming. Review of the SAF during the visit was a natural progression of the visit and using different team members to review CR helped with time efficiency.

## Discussion/conclusion

We successfully implemented a method to identify and address SDOH during the clinical encounter. Nearly, 600 patients completed the SAF during the pilot study. Our pilot project found that in our urban primary care setting between a quarter and a third of patients surveyed had at least one SDOH and in one-third of these positive surveys patients wanted to discuss the SDOH with their provider. Moreover, a majority of the positive forms had more than one SDOH marked, with 28% having three or more SDOH marked. Our findings are consistent with other studies regarding frequency of SDOH. A similar study in another urban primary care clinic found that 27% of patients had some SDOH impacting their health [4]. Outside of health, the most frequent patient-identified factors impacting their health were financial issues, food insecurity, transportation issues, housing insecurity, and employment. Prior to this pilot, there was no routine assessment in our clinic to screen for SDOH during patient check-in. The SAF was quick, simple to administer, and easy for the physician to review. They were often completed by the time the provider entered the room. Unlike longer screeners such as PRAPARE, this screener is short and uses graphics as well as words, which may be easier for those with lower literacy levels to use. The online CR tool was quickly accessible in the primary care setting to address concerns.

Although one-third of patients who did identify at least one SDOH wanted to discuss it with their provider, a majority did not want to discuss these issues with their healthcare team. The reason for this is unclear and not identifiable with our data. However, recent research suggests that although patients do want their healthcare team to screen for SDOH, about 40% are wary of this data collection and sharing of this data [6,7]. This may be due to concerns about stigma from other healthcare providers seeing a SDOH on their problem list.

Our study did face some limitations. This was a small study looking at just two providers’ patients in a large group primary care practice. The exact demographic makeup of our pilot participants is unknown; a future study could gather demographic characteristics as well as capture more patient-specific data including insurance information to better assess SDOH needs. While we obtained staff feedback and provided staff training prior to the implementation of the SAF, we did not establish a feedback loop to assess the impact on clinic staff and patients. Follow-up studies will include this additional step.

The SAF was anecdotally difficult to understand for some patients; particularly the “health” graphic was often misinterpreted as the reason for visit which may falsely elevate the actual SDOH incidence. Removal of “health” from data analysis did reduce the total number of positive screens by 6%. Future studies will need to refine the survey to make it more understandable and ensure the correct data is being captured.

We were not able to easily capture use of CR or referral to community resources and follow up on these referrals. This will be an important area for future research to better determine the impact of addressing SDOH in the primary care setting on health outcomes.

Lastly, our study has limited external validity because CR is not widely available to all regions of the USA and therefore is not easily replicated.

SDOH are critical factors to achieve improved health outcomes, improved quality of life, and improved community health. The SAF allowed us to easily and quickly unveil unique struggles and identify barriers to care, allowing for more patient-centered medical care. Primary care is an optimal setting to use the SAF to gather important information about the patient to guide healthcare decisions and identify needed supports. Overall, this short screener identified a similar frequency of SDOH as larger surveys had, indicating it may be an accurate tool to reliably identify SDOH to then guide support in a primary care setting.

## Supporting information

Kalsi et al. supplementary material Kalsi et al. supplementary material

## Supplementary material

The supplementary material for this article can be found at https://doi.org/10.1017/cts.2024.511.
