# Supplementary material for: Identifying and addressing social determinants of health to improve patient-centered care
Source: J Clin Transl Sci. 2024 Apr 5;8(1):e73. doi: 10.1017/cts.2024.511 (PMC11058574; doi:10.1017/cts.2024.511)
Supplement: Kalsi et al. supplementary material [file S2059866124005119sup001.pdf]

SOCIAL ASSESSMENT FORM

This form helps us identify any issues, concerns or challenges that may be impacting your health and wellbeing. You will have the option to discuss your responses with your health-care team. **Regardless of whether or not you choose to discuss your responses with your provider, we encourage you to visit CommunityRelay.com.** Community Relay is a free, confidential resource that enables you to search for free- or reduced-cost programs and services by zip code.

☐ **I prefer not to complete this form.**

---

1 Please mark any of the following areas that may be impacting your health and wellbeing, check all that apply.

☐ **FOOD**<br>Food pantries, nutrition, meal planning	☐ **HOUSING**<br>Temporary housing, repairs, financial assistance	☐ **GOODS**<br>Household items, toys, clothing, medical supplies	☐ **HEALTH**<br>Nutrition, medical care, vision care	☐ **FINANCIAL**<br>Financial education, tax preparation, insurance
☐ **TRANSIT**<br>Bus passes, financial assistance for gas or public transportation	☐ **CARE**<br>End of life care, daytime care, residential care	☐ **EDUCATION**<br>Preschool, screening and exams, financial assistance	☐ **EMPLOYMENT**<br>Job placement, workplace rights, skills and training	☐ **LEGAL**<br>Mediation, representation, interpretation

☐ None of the above apply to me today.

2 Please share any supporting information and/or additional **non-medical** concerns:

3 Would you like to discuss your responses with a member of your health-care team?

☐ **YES** ☐ **NO**

VISIT COMMUNITY RELAY

© Community Relay, 2022
